# Coherent scatter-controlled phase-change grating structures in silicon using femtosecond laser pulses

**DOI:** 10.1038/s41598-017-04891-3

**Published:** 2017-07-04

**Authors:** Yasser Fuentes-Edfuf, Mario Garcia-Lechuga, Daniel Puerto, Camilo Florian, Adianez Garcia-Leis, Santiago Sanchez-Cortes, Javier Solis, Jan Siegel

**Affiliations:** 1 Laser Processing Group, Instituto de Óptica, IO-CSIC, Serrano 121, 28006 Madrid, Spain; 2 Instituto de Estructura de la Materia, CSIC, Serrano 121, 28006 Madrid, Spain

## Abstract

Periodic structures of alternating amorphous-crystalline fringes have been fabricated in silicon using repetitive femtosecond laser exposure (800 nm wavelength and 120 fs duration). The method is based on the interference of the incident laser light with far- and near-field scattered light, leading to local melting at the interference maxima, as demonstrated by femtosecond microscopy. Exploiting this strategy, lines of highly regular amorphous fringes can be written. The fringes have been characterized in detail using optical microscopy combined modelling, which enables a determination of the three-dimensional shape of individual fringes. 2D micro-Raman spectroscopy reveals that the space between amorphous fringes remains crystalline. We demonstrate that the fringe period can be tuned over a range of 410 nm – 13 µm by changing the angle of incidence and inverting the beam scan direction. Fine control over the lateral dimensions, thickness, surface depression and optical contrast of the fringes is obtained via adjustment of pulse number, fluence and spot size. Large-area, highly homogeneous gratings composed of amorphous fringes with micrometer width and millimeter length can readily be fabricated. The here presented fabrication technique is expected to have applications in the fields of optics, nanoelectronics, and mechatronics and should be applicable to other materials.

## Introduction

Direct writing of structures on material surfaces using focused laser beams is a powerful contact-less patterning technique that is widely employed in industry for numerous applications. One of the biggest advantages relies on the possibility to modify virtually any material in a fast and flexible way, starting from a digital design towards the fabricated structure, without the need of masks or master samples. However, there are some challenges concerning the spatial resolution and the processing time over large areas: excepting super-resolution approaches^1^, the diffraction limit of light imposes a fundamental barrier for the minimum achievable feature size. For instance, the use of shorter wavelengths (i.e. UV) and high numerical aperture optics becomes essential for modifying the material with nanometric spatial resolution. Under such conditions of tight focusing for writing each individual feature sequentially, processing times become prohibitively long when large areas need to be fabricated.

An alternative to laser direct-writing is a parallel laser patterning strategy that exploits self-organization processes triggered in the material, which allows the structuring of materials with high spatial resolution using weakly focused laser beams. Under certain conditions, the laser irradiation can trigger self-assembly processes at the material surface, which lead to the formation of so-called laser-induced periodic surface structures (LIPSS)^2–10^. The process is generally understood as caused by interference of the incident laser light with a surface wave scattered/coupled at surface roughness^11^. However, there is a vivid debate about the exact mechanism, whether it is dominated by surface plasmon polariton propagation or optical near- and far-field scattering^12–15^. Regardless of the physical origin, the periodic intensity modulation gradually evolves upon multiple pulse irradiation and an equivalent periodic surface modulation is finally imprinted in the material. While in most cases the process leads to local ablation or material reorganization, we reported recently a proof-of-principle of a fabrication process based on the local amorphization of crystalline silicon, leading to amorphous-crystalline LIPSS using laser fluences below the ablation threshold^15, 16^. Employing laser beam scanning at different wavelengths it was shown that parallel amorphous fringes could be written by a proper adjustment of laser fluence, repetition rate and scan speed.

In the present work, we exploit the full potential of this fabrication technique by controlling systematically the laser irradiation conditions, demonstrating full control over their final properties such as fringe period, size, thickness, surface topography, lateral extension and optical contrast. Moreover, we provide a complete characterization of the structures, using optical microscopy, femtosecond microscopy, micro-Raman spectroscopy and scanning electron microscopy, which allows a 3D reconstruction of individual fringes and yields important insights on their formation process.

## Results and Discussion

### Structures fabricated at normal incidence (*θ* = *0°*)

**Figure 1:**
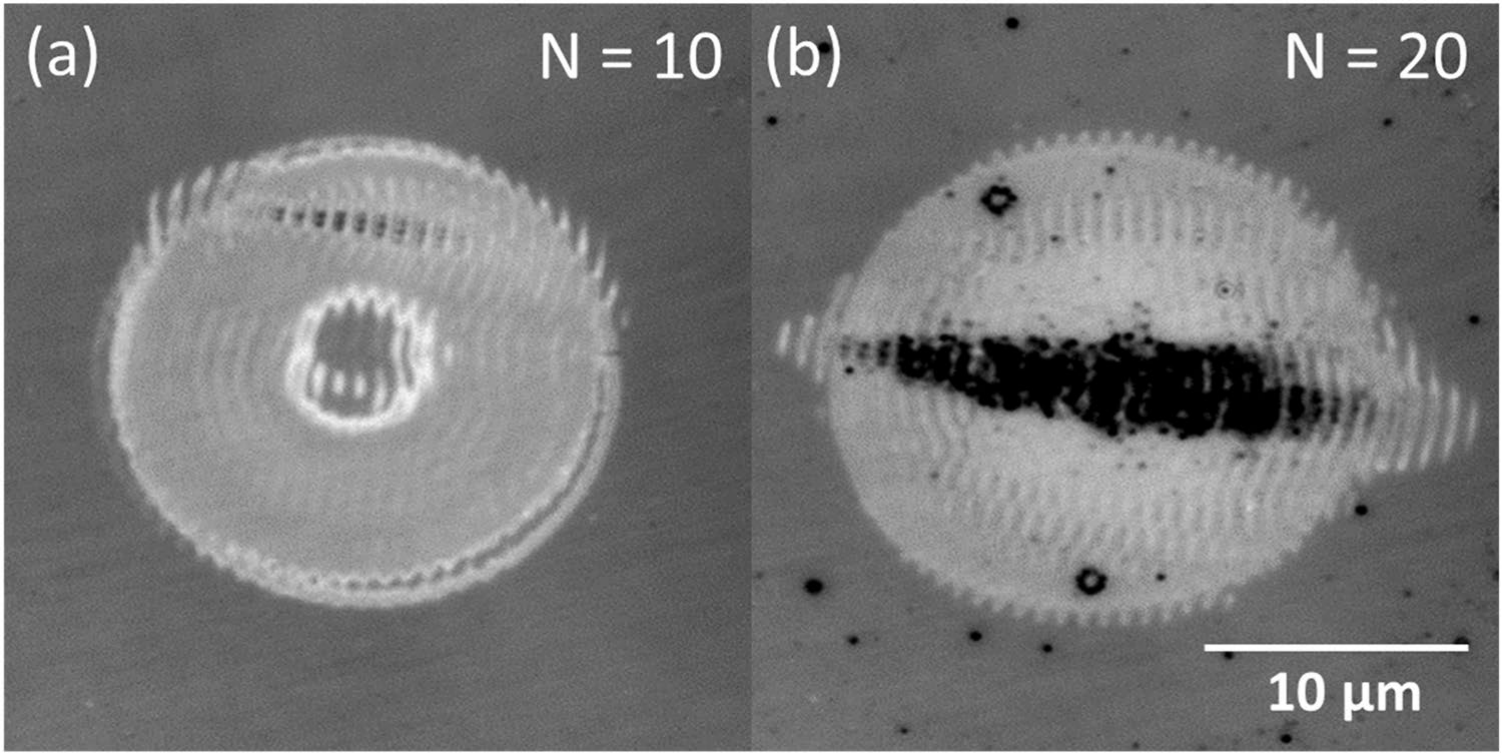
Optical micrographs of the Si surface after irradiation with (**a**) *N* = *10* and (**b**) *N* = *20* horizontally polarized laser pulses at normal incidence (*θ* = *0°*) and *F* = *270* *mJ*/*cm* ^2^.

Figure 1 shows optical microscopy images of the Si sample after exposure to a different number N of laser pulses at a constant fluence *F* = *270* *mJ*/*cm* ^2^. For *N* = *10* (c.f. Fig. 1(a)), an annular structure can be observed, featuring a ring of increased reflectivity and a central disk of similar reflectivity as the non-exposed region. The reflectivity increase within the annular region is consistent with surface amorphization as reported by Bonse^17, 18^, since the absorption coefficient of the amorphous phase of Si at the illumination wavelength is known to be much higher than that of the crystalline phase^19^, leading to a reflectivity increase. Superimposed to this relatively large structure, parallel bright fringes can be appreciated, which are oriented vertical and thus perpendicular to the direction of the laser polarization. This is a characteristic of the so-called low spatial frequency (LSF) LIPSS. As the pulse number increases, a progressive darkening, indicative of surface ablation, is observed in the central region, extending beyond the amorphous region for *N* = *20* (c.f. Fig. 1(b)). A similar behavior of amorphous fringe formation and transition to ablation has been reported upon irradiation with 370 fs, 1030 nm laser pulses, although in that case it was at lower N-values, leading to a better fringe homogeneity and alignment^16^. The non-circular shape of the ablation region is a consequence of the directional surface wave triggered at the fringe structures, which is more efficient along the direction of the laser polarization vector, leading to a horizontal enlargement of the ablated region^20^. Although SPPs can theoretically only be coupled and propagate at a dielectric/metal interface, laser-induced free-electron generation in semiconductors and dielectrics turns them transiently into a metal-like state, generating conditions for SPP propagation^12^. Yet, there is still a vivid debate about other mechanisms that contribute to the formation of LIPSS in semiconductors, amongst which optical near- and far-field scattering process have recently been identified^14, 15^.

**Figure 2:**
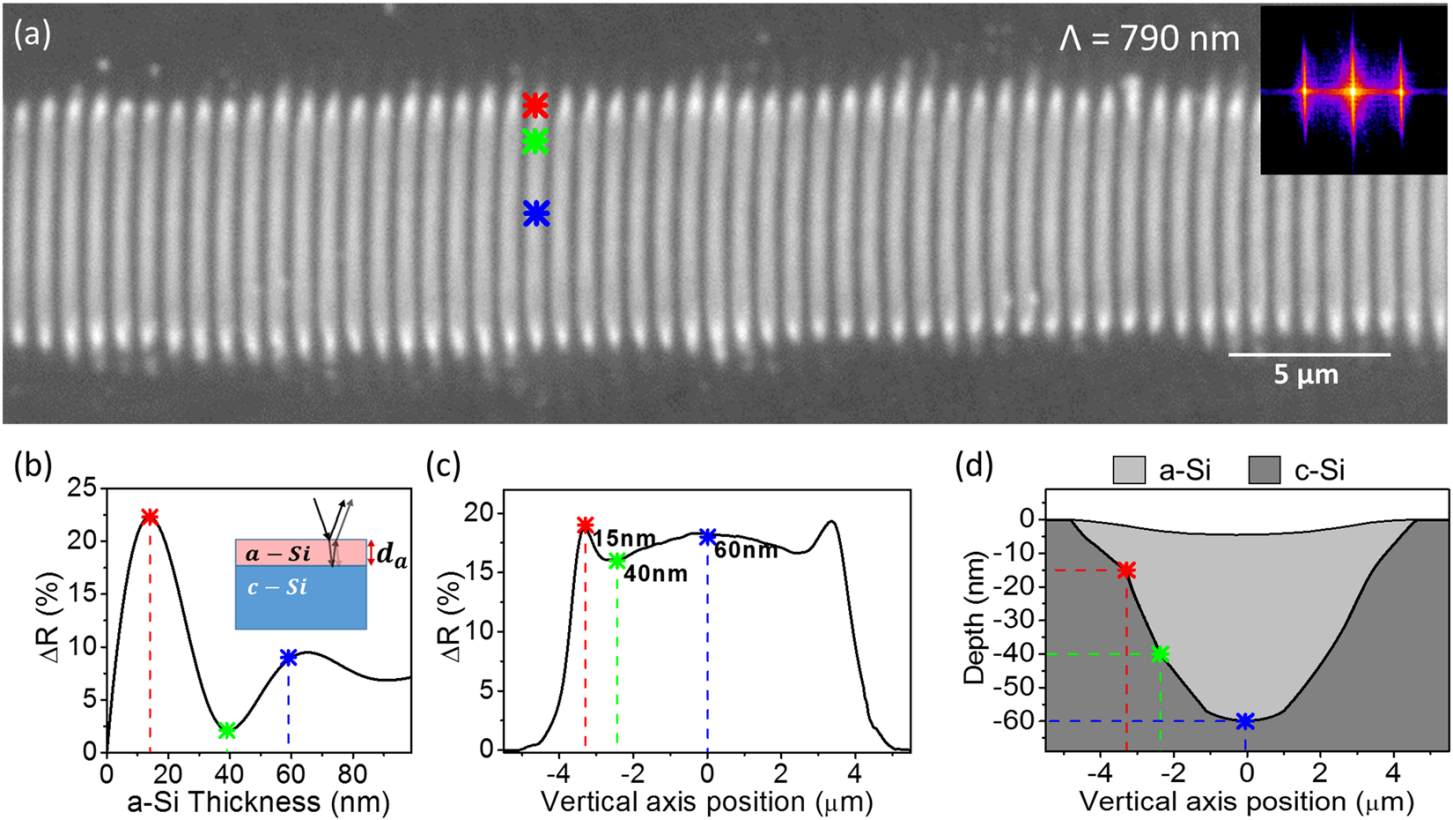
(**a**) Optical micrograph of extended amorphous-crystalline fringes in Si after irradiating the sample with horizontally polarized laser pulses at 100 Hz moving the sample at constant speed *v* = *200* μm/s, using *F* = *190* *mJ*/*cm* ^2^. Inset: Two-dimensional Fourier transform of (**a**). The first order corresponds to the value of the period *Λ* indicated. (**b**) Calculated reflectivity change as a function of thickness of an amorphous top layer, as indicated in the inset and using the model described in the text. The inflection points of the curve are marked by star symbols. (**c**) Experimental vertical profiles of (**c**) the reflectivity change and (**d**) the surface topography (top curve in (**d**)) of a single fringe shown in (**a**). The lower curve in (**d**), indicating the thickness of the amorphous layer has been extracted from (**b**) and (**c**), effectively displaying the cross section of an amorphous fringe (shaded area).

A powerful method to improve fringe homogeneity and alignment, as well as to extend fringes in 2D, is to scan the laser spot over the surface at a well-defined repetition rate and speed^16^. Figure 2(a) shows the best result for a structure written in Si while moving the sample, under similar conditions as those used in Fig. 1(a), although a considerable reduction of fluence (*F* = *190* *mJ*/*cm* ^2^) was necessary to prevent ablation. The well-defined periodicity of the structure can be appreciated in the fast Fourier transform performed on the micrograph, featuring a period *Λ* = *790* ± *30* *nm*. This value is close to the laser wavelength, consistent with conventional LSF-LIPSS structures in Si^13^. Figure 2(c) shows the reflectivity profile through a single fringe (drawn vertically across the structure shown in Fig. 2(a)). As demonstrated for single pulse irradiation of Si〈111〉^17, 18^, the thickness of the amorphous surface layer formed can be estimated by modeling a multilayer system based on Abèles theory using the optical constants of amorphous and crystalline silicon^19^. We have applied this approach to the determination of the thickness of a single amorphous fringe. The result of the calculation is shown in Fig. 2(b), representing the calculated reflectivity change as a function of thickness of the amorphous top layer. The oscillatory behavior of the curve is caused by interference of the light reflected at the two interfaces and the damping behavior is due to the elevated absorption coefficient of the amorphous phase.

Taking into account the relation between reflectivity and thickness of the amorphous layer shown in Fig. 2(b), the layer thickness at certain local positions of an individual fringe in Fig. 2(a) can be determined. Non-ambiguous reflectivity values correspond to the inflection points of the calculation (first maximum *I*_*1*_, first relative minimum *I*_*2*_, second relative maximum *I*_*3*_) and correspond to the thickness values *d*_*1*_ = *15* *nm*, *d*_*2*_ = *40* *nm* and *d*_*3*_ = *60* *nm*, which are included in Fig. 2(c).

Topography measurements have been performed on the structure shown in Fig. 2(a). From the extracted topography profile (included in Fig. 2(d)) a maximum surface depression of 5 nm can be observed. It has to be mentioned that we have multiplied the experimentally measured height value by a factor of two due to the fact that the optical profiler does not resolve individual fringes laterally (see experimental section) and therefore yields an average of fringe/no fringe. Combining the topography profile and the extracted thickness of the amorphous layer at different positions (c.f. Fig. 2(c)) it is straightforward to reconstruct the cross section of an amorphous fringe, as shown in Fig. 2(d).

### Structures fabricated at oblique incidence (*θ* = *52°*)

A simple way to change the period of LIPSS is to use oblique incidence upon irradiation. An angle dependence of LSF-LIPSS in static irradiation experiments has been first observed by Young *et al*.^21^, reporting two possible periods for p-polarized light, according to *Λ* ^*s*−^ = *λ*/*(1* + sin *θ)* and *Λ* ^*s*+^  = *λ*/*(1* − sin *θ)*, derived from a purely geometrical scattering model. Both periods were found to co-exist in the same area in form of a superposition of them. To the best of our knowledge, none of the studies by other groups reporting angle dependence of the period^21–24^, have presented a convincing strategy to preferentially select one or the other period. In our recent publication on the formation dynamics of amorphous-crystalline fringes, we report that preferential selection of the period to be imprinted is possible by a convenient choice of the scanning direction^15^. In the present paper, we explain this effect and identify and investigate the additional parameters that are important to control the physical dimensions of the fringe structures formed, thus obtaining full control over the period, width, length and thickness of the fringes.

**Figure 3:**
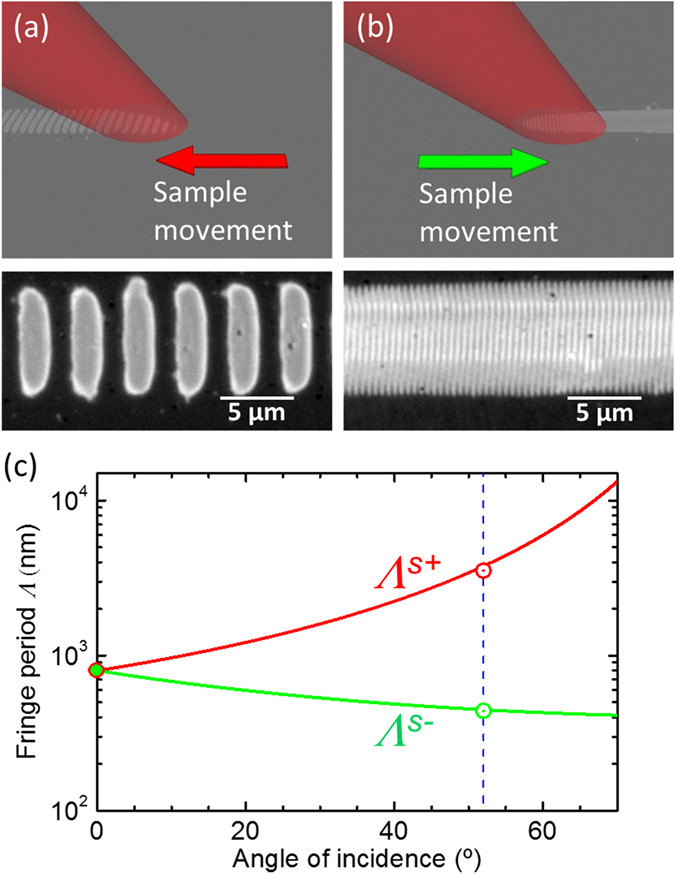
(**a**,**b**) Sketches of the strategies to preferentially write (**a**) *Λ* ^*s*+^ or (**b**) *Λ* ^*s*−^ LIPSS based on selecting the scan direction accompanied by optical micrographs of the written structures. Experimental parameters: *θ* = *52°*, *F* = *145* *mJ*/*cm* ^2^, *v* = *200 µm*/*s* (**c**) Calculation of the fringe periods *Λ* ^*s*+^ and *Λ* ^*s*−^ obtainable for a single laser wavelength *(λ* = *800 nm*) as a function of angle of incidence. The symbols correspond to the experimental values obtained at *θ* = *0°* and *52°*.

Figure 3 illustrates how by inverting the direction of sample movement the imprinted period can be changed from *Λ* ^*s*+^ to *Λ* ^*s*−^. Figure 3(a) shows a sketch for the case of a sample moving to the left and a laser beam incident at an angle *θ* from the left. Consequently, the forward-scattered light and its interference with directly incident light on unexposed regions leads to “priming” the sample with period *Λ* ^*s*+^, which is then effectively imprinted upon sample movement. In the case of opposite sample movement (Fig. 3(b)) it is the backscattered light and its interference with direct light on unexposed regions that leads to “priming” and imprinting with period *Λ* ^*s*−^. The corresponding experimental results shown below each sketch correspond to identical writing parameters except for the sample movement direction. The experimentally obtained periods *Λ* ^*s*+*,exp*^ = *3.53 *±* 0.20 µm* and *Λ* ^*s*−*,exp*^ = *440* ± *20* *nm* agree well with the values calculated from the simple scatter model for (*θ* = *52°*) *Λ* ^*s*+*,theo*^ = *3.78 µm* and *Λ* ^*s*−*,theo*^ = *447 nm*, as shown in Fig. 3(c).

This ability, to preferentially select the LIPSS period by a proper choice of scan direction, has enormous technological potential. As can be seen in Fig. 3(c), the range of periods that can be imprinted with a single laser wavelength (*λ* = *800* *nm*) is vast, covering *Λ* = *410* *nm* − *13* *µm* for angles up to *θ* = *70°*. By frequency doubling the output of the same laser system, a standard option in ultrafast laser sources, this range can be easily extended to shorter periods (*Λ* = *205* *nm* − *6.5* *µm*).

**Figure 4:**
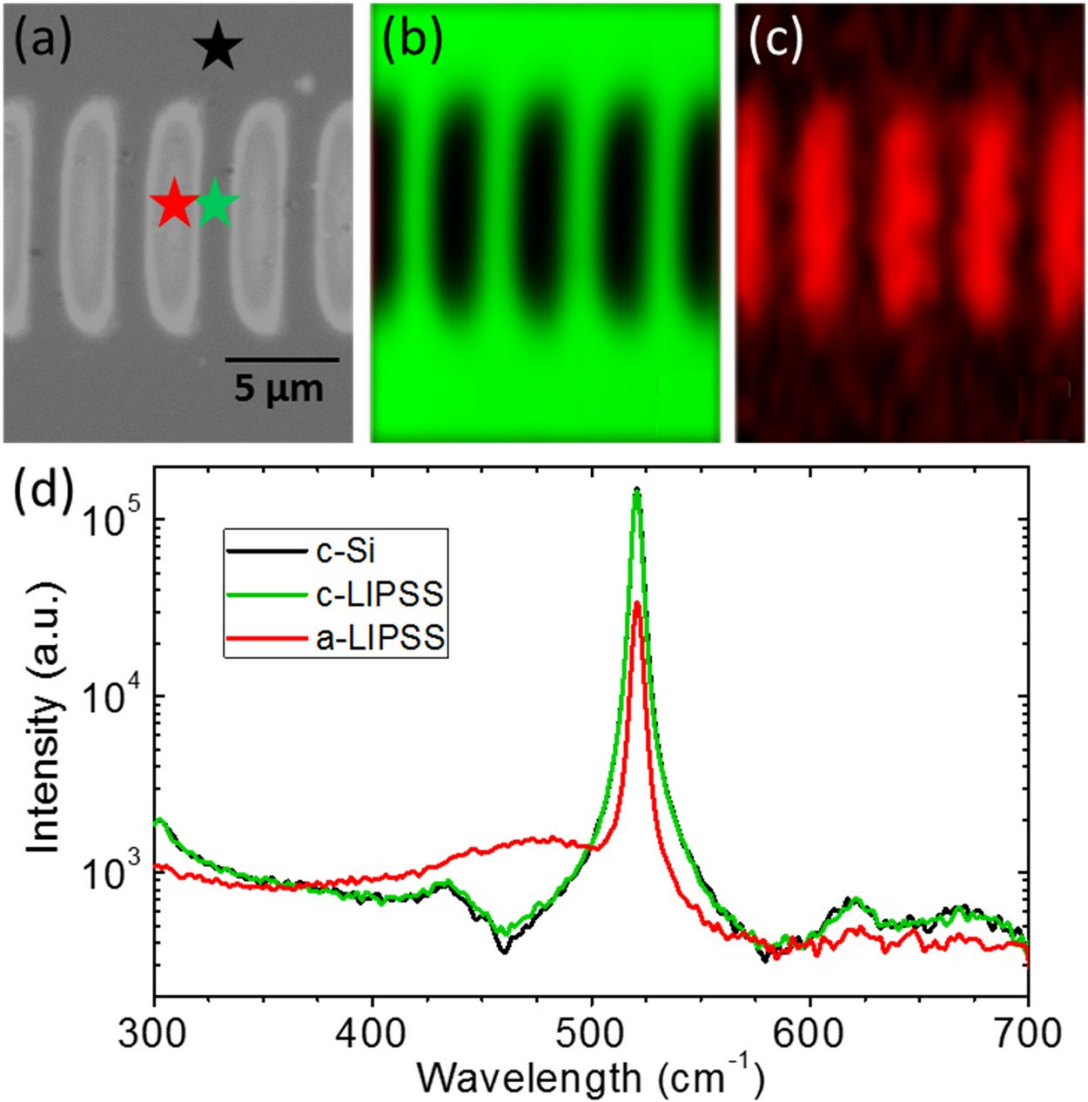
Raman spectroscopy of long-period amorphous fringes (*Λ* ^*s*+^ LIPSS). (**a**) Optical micrograph of the studied region. The symbols mark the positions at which the point measurements shown in (**d**) were performed. (**b**) Map of the signal amplitude of the Raman band of c-Si centered at 520.5 cm^−1^. (**c**) Map of the signal amplitude of the Raman band of a-Si at 473 cm^−1^. (**d**) Raman spectra recorded at the positions marked in (**a**).

We have studied in more detail the fringes produced under oblique incidence. Micro-Raman spectroscopy is a suitable high-sensitivity technique to detect the presence of the amorphous phase in laser irradiated crystalline silicon, even if present only in form of a thin surface layer^18^. Yet, the short LIPSS period, close to the laser wavelength for normal incidence, poses a challenge to the spatial resolution of micro-Raman spectroscopy, which effectively averages over fringe and inter-fringe space^16^. Here, taking advantage of the fact that the fringe period can be increased by changing the angle of incidence, we have performed micro-Raman spectroscopy on a single *Λ* ^*s*+^ fringe, at the position indicated in Fig. 4(a). The resulting spectrum, shown in Fig. 4(d), features the presence of the broad band centered at 473 cm^−1^, which is the fingerprint of amorphous silicon. We have also recorded spectra between two fringes and in an unexposed region, at the positions marked in Fig. 4(a). Both spectra are included in Fig. 4(d) and are almost identical, featuring only the characteristic strong peak of crystalline silicon, centered at 520.5 cm^−1^, which allows the conclusion that the inter-fringe region remains crystalline. The fact that the spectrum in the fringe region still shows a strong contribution from c-Si is caused by the shallow thickness of the amorphous surface layer compared to the much larger depth of focus of the confocal Raman microscope, effectively collecting most Raman signal from the crystalline regions underneath the amorphous layer. Using modern Raman microscopes it is also possible to map entire regions, rather than performing single point studies. Figure 4(b,c) show Raman maps of the amplitude of the crystalline and amorphous peak of the region shown in Fig. 4(a). The images nicely confirm the amorphous nature of the fringes and the crystalline nature of the inter-fringe region.

**Figure 5:**
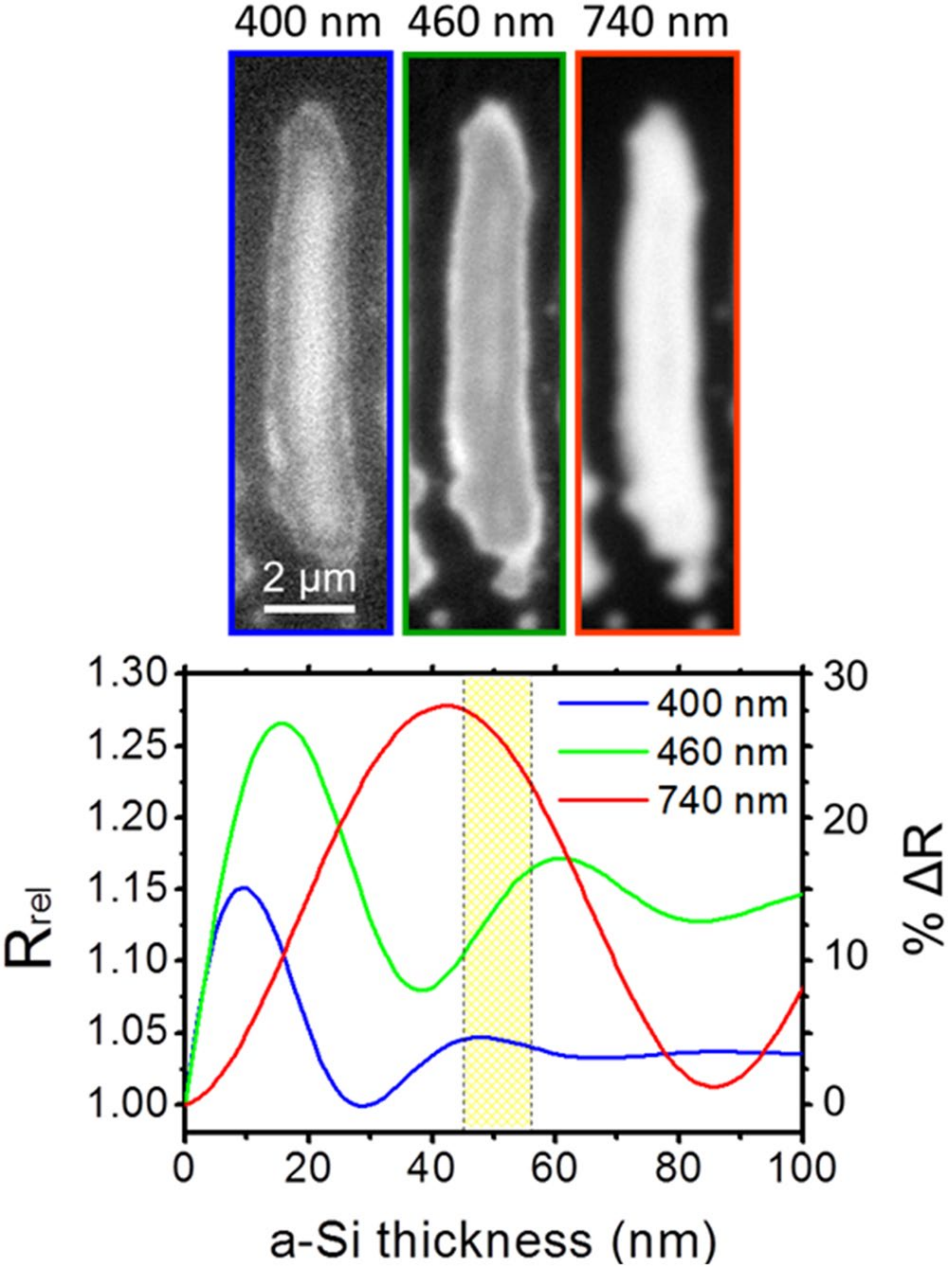
Optical study of a single long-period amorphous fringe (*Λ* ^*s*+^ LIPSS) for thickness determination. Top row: Optical micrographs of the same fringe recorded upon illumination at different light wavelengths (see labels). Bottom graph: Calculated reflectivity change as a function of thickness of an amorphous top layer for the three illumination wavelength employed, using the model described in the text. The shaded area corresponds to the interval for the maximum thickness, determined from a comparison of reflectivity data and calculation.

As discussed in the previous section, the thickness of the amorphous layer can be determined by measuring the spatial reflectivity modulation upon illumination with monochromatic light, comparing it to the calculation obtained by a multilayer model^17, 18^. Exploiting the larger width of the *Λ* ^*s*+^ fringes, we have tested this approach by using different illumination wavelengths, which should lead to a wavelength-dependent change of the modulation. Figure 5(a) shows optical micrographs of a single *Λ* ^*s*+^ fringe illuminated at 400 nm, 460 nm and 760 nm. It is immediately evident that the fringe appearance is strongly wavelength-dependent. In particular, the images recorded at short wavelengths (400 nm and 460 nm) feature contours of different grey levels inside the fringe, passing through maxima and minima. The relatively large fringe width allows resolving this oscillation, which corresponds to an increase of the thickness of the amorphous layer towards the fringe center. The corresponding calculations for these wavelengths are plotted in Fig. 5. In contrast, the image recorded at the longer wavelength (760 nm) does not show an oscillation but only a strong reflectivity increase. This is consistent with the corresponding calculation. By means of this approach, combing illumination at different wavelength and modelling it is possible to determine quite precisely the thickness profile of a single fringe, yielding a maximum thickness value in the fringe center of d = 51 ± 5 nm, as indicated in the plot in form of the shaded area.

**Figure 6:**
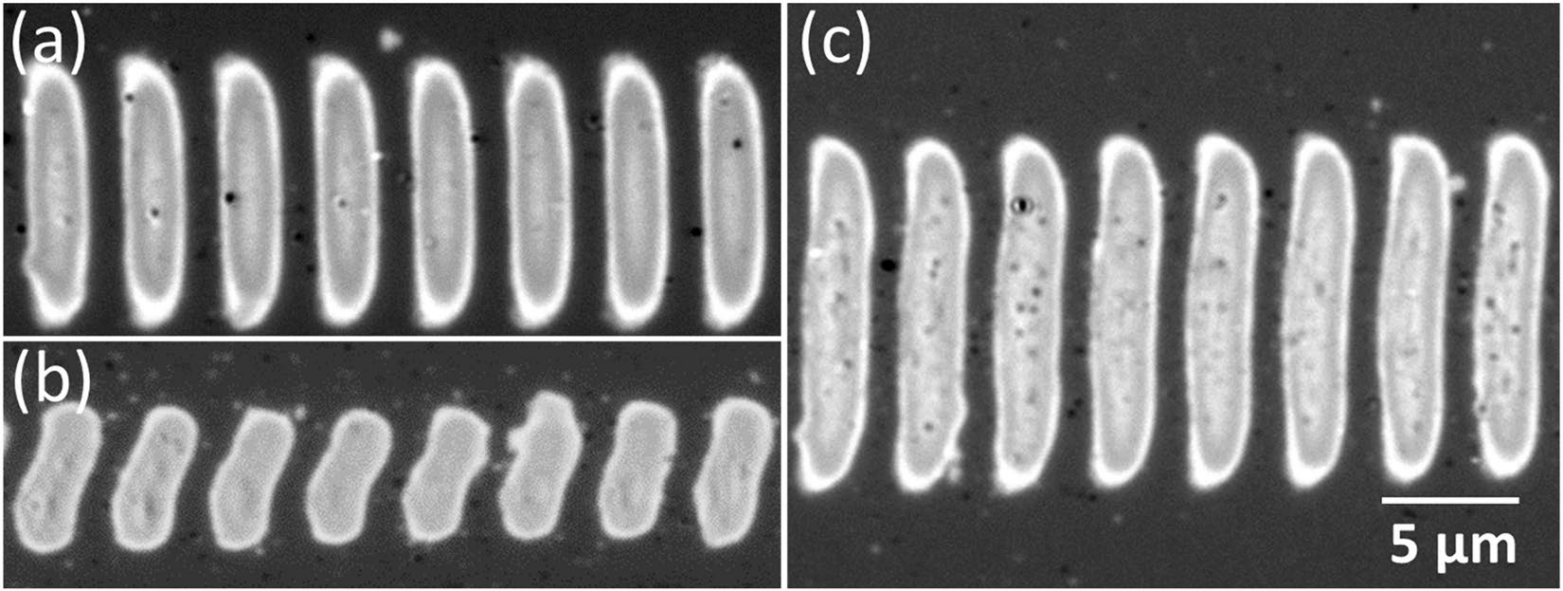
Optical micrographs of amorphous fringes with constant period *Λ* ^*s*+^ but different length, obtained by changing spot diameter *d* and laser fluence *F*. (**a**) *d*_*y*_ = *59* *µm*, *F* = *146* *mJ*/*cm* ^2^, *v* = *360* *µm*/*s* (**b**) *d*_*y*_ = *22* *µm*, *F* = *190* *mJ*/*cm* ^2^, *v* = *100* *µm*/*s* (**c**) *d*_*y*_ = *59 µm*, *F* = *148* *mJ*/*cm* ^2^, *v* = *360* *µm*/*s*.

The in-plane aspect ratio of the fringes can be controlled by means of three parameters, namely laser spot size, fluence, and pulse number. The laser spot diameter has a direct effect, leading to a corresponding change in fringe length, maintaining width and period. This can be seen in Fig. 6(b), in which a spot size reduction by a factor *C*_*spot*_ = *2.7* compared to the one used for Fig. 6(a) (by means of changing both, the focal length of the lens and beam diameter) leads to change in fringe length by a factor of *C*_*length*_ = *1.9*. The fact that *C*_*spot*_ *≠* *C*_*length*_ is caused by the dependence of the aspect ratio on the other two parameters, which are slightly different in (a) and (b). The influence of the laser fluence on fringe length can be seen in Fig. 6(c), obtained with exactly the same parameters as Fig. 6(a) but at slightly higher fluence (by only 1.3%, i.e *C*_*fluence*_ = *1.013*). Yet, the resulting increase in fringe length is pronounced (*C*_*length*_ = *1.4*), illustrating the fact that the writing process is performed near threshold.

A study of the influence of the pulse number on the fringe length is relatively complex, as it requires a simultaneous compensation of the incident laser fluence in order to guarantee fringe formation but avoiding surface ablation, which would occur for elevated pulse numbers. We have used the definition used in ref. ^16^ to determine the effective pulse number per unit area for a given spot size and sample speed. To this end we use the vertical length of the optimum fringe structure formed for a given laser spot size (exploring laser fluence and scan speed for optimization) as criteria for defining an ‘effective vertical spot diameter’ (d_eff_) for each case. The values obtained are *d*_*eff, small*_ = *9.8* *μm* and *d*_*eff, large*_ = *17.4* *μm*, which are much smaller than the corresponding Gaussian spot diameters *d*_*y, small*_ = *22* *μm* and *d*_*y, large*_ = *59* *μm*. They represent a fluence threshold (*F*_*amorph*_) of around *0.94* × *F*_*peak fluence*_. The effective pulse number has then been calculated as *N*_*eff*_ = *d*_*eff*_ × *100* *Hz*/*v*, with *100* *Hz* being the laser repetition rate and *v* the scan speed.

**Figure 7:**
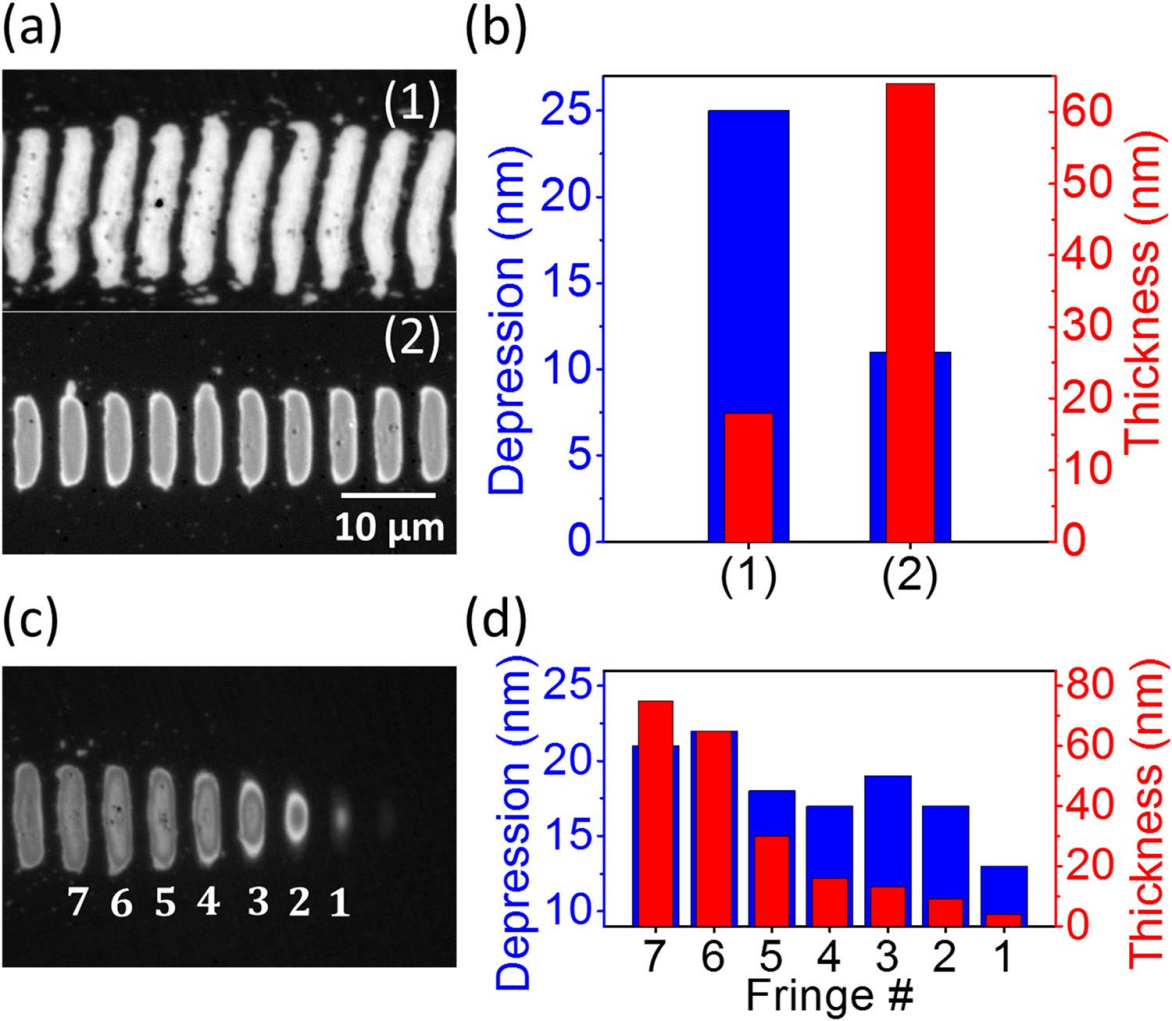
(**a**) Optical micrograph of two lines of *Λ* ^*s*+^ fringes written with different effective pulse numbers *N*_*eff*_. For high N_eff_ values the fluence was reduced to prevent ablation. (1) *N*_*eff*_ = *158*, *F* = *120* *mJ*/*cm* ^2^, (2) *N*_*eff*_ = *55*, *F* = *140* *mJ*/*cm* ^2^. (**b**) Representation of the surface depression and thickness of the fringes shown in (**a**). (**c**) End of a line of fringes written. The effect of progressive fringe length shrinking is caused by closing the laser shutter, while the sample was moving from right to left. (**d**) Representation of the surface depression and thickness of the fringes shown in (**c**).

Figure 7(a) shows two lines of fringes written with different effective pulse numbers. In order to prevent ablation of the structure written at high pulse number, the fluence was lowered for that case. While the expected difference in the obtained fringe length can indeed be observed, more striking is the strong difference in fringe brightness, indicative of a different thickness of the amorphous layer of both fringe lines. Employing the calibration curve shown in Fig. 5 for the illumination wavelength used (460 nm) we have determined the thickness values and displayed in Fig. 7(b), which also shows the values of surface depression measured by an optical profiler. At low fluence and high pulse numbers a shallow thickness but considerable surface depression is obtained. The opposite behavior is observed at higher fluence and lower pulse numbers. Two conclusions can be drawn at this point: First, an elevated fluence favors the formation of a thick amorphous layer as it leads to deep melting. Second, high pulse numbers lead to an increased surface depression, most likely due to progressive evaporation, as reported by Tsibidis *et al*.^25^. It has to be taken into account that the energy deposition increases with pulse number since the progressively growing amorphous layer enhances absorption, leading to high temperatures and surface evaporation.

Interesting information about the influence of the pulse number and local fluence can be obtained from an experiment in which the shutter of the laser is closed during the LIPSS writing process while moving the sample. The effect on the structure is, as shown in Fig. 7(c), that the end of the line of fringes narrows down in the vertical dimension before the fringes disappear. The underlying reason is that fringe 1 (see labels in Fig. 7(c)) has been exposed to fewer pulses than fringe 7 that has already traversed the center of the focal region. For the parameters used (100 Hz repetition rate and 0.2 mm/s speed) and assuming an instantaneous shutter closure, fringe 1 has received 14 pulses less than fringe 7. This experiment allows therefore to study in detail the influence of the pulse number by analyzing each fringe individually. From Fig. 7(d) can be concluded that a progressive decrease in surface depression and thickness of the amorphous layer occurs from fringe 7 down to fringe 1, demonstrating the fine control that can be exerted on both features by adjusting the pulse number.

**Figure 8:**
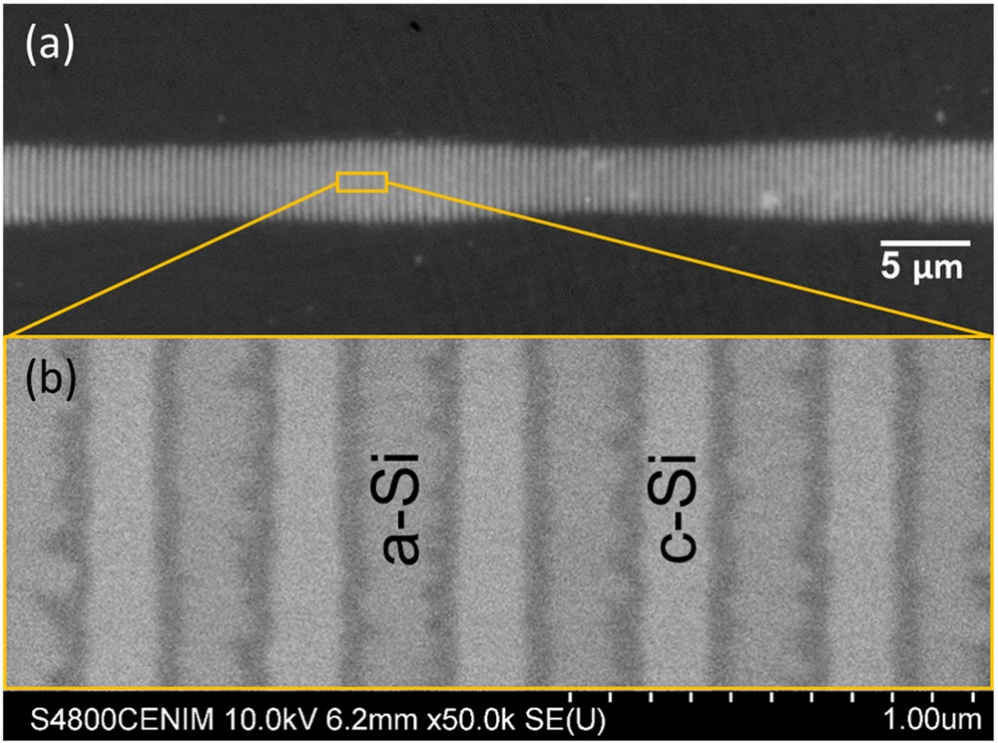
Line of short-period amorphous fringes (*Λ* ^*s*−^ LIPSS) written at *F* = *180* *mJ*/*cm* ^2^ and *320* *µm*/*s*. (**a**) Optical micrograph. The rectangle marks the region shown in (**b**). (**b**) SEM image. The amorphous and crystalline regions are marked by “a-Si” and “c-Si”, respectively.

The second fringe type, *Λ* ^*s*−^ LIPSS produced upon inversion of the scan direction, have also been studied in more detail. Figure 8(a) shows a typical line of fringes produced, featuring a very short period *Λ* = *440* ± *20* *nm*, which is barely resolvable with optical microscopy. The slight modulation in the envelope of the fringe length is probably caused by slight fluctuations in laser intensity, which have a strong impact when working near the amorphization threshold.

Inspection with a scanning electron microscope provides higher resolution images, as shown in Fig. 8(b). Using secondary electron detection, contrast between the amorphous and crystalline phase can be observed despite the shallow thickness of the amorphous layer compared to the considerable penetration depth of electrons. Yet, the contrast is different and should not be compared to the one found in the optical micrographs. The higher spatial resolution of the SEM image allows resolving the sharpness of the boundary between crystalline and amorphous regions. The boundary is very sharp, which is consistent with the formation process based on melting above a threshold. The image also reveals that fringes are not interconnected, despite the short period. This finding is important as it demonstrates that the fringe period can still be further downscaled (e.g. using larger angles or shorter wavelengths, as illustrated in Fig. 3(c)).

**Figure 9:**
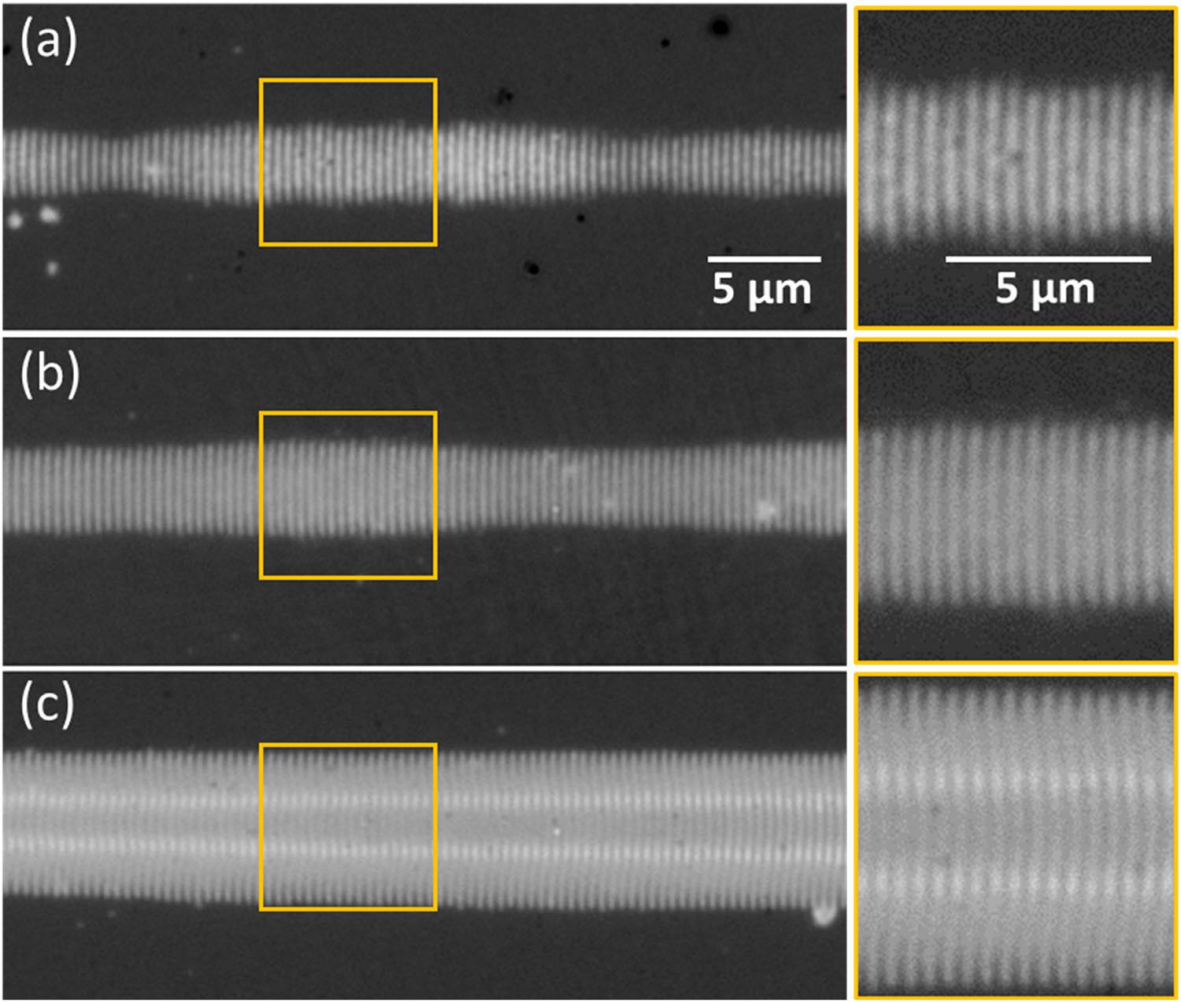
Optical micrographs of lines of short-period amorphous fringes (*Λ* ^*s*−^ LIPSS) written at constant fluence (*F* = *180* *mJ*/*cm* ^2^) but different scanning speeds (**a**) *v* = *320* *µm*/*s*, *N*_*eff*_ = *33* (**b**) *v* = *300* *µm*/*s*, *N*_*eff*_ = *35* (**c**) *v* = *250* *µm*/*s*, *N*_*eff*_ = *42*.

Like for long-period fringes, we have studied the influence of the effective pulse number also on the formation of *Λ* ^*s*−^ LIPSS. Figure 9 shows three lines written under the same conditions, except scan velocity, which determines the effective pulse number. A considerable increase in linewidth (almost factor two) is observed when increasing from *N*_*eff*_ = *33* (Fig. 9(a)) to *N*_*eff*_ = *42* (Fig. 9(c)). Moreover, the reflectivity along the vertical dimension of the fringes, which is initially constant, shows a modulation that indicates a change in thickness of the amorphous layer (see the central part in Fig. 9(c)).

### Formation mechanism of fringe structures

**Figure 10:**
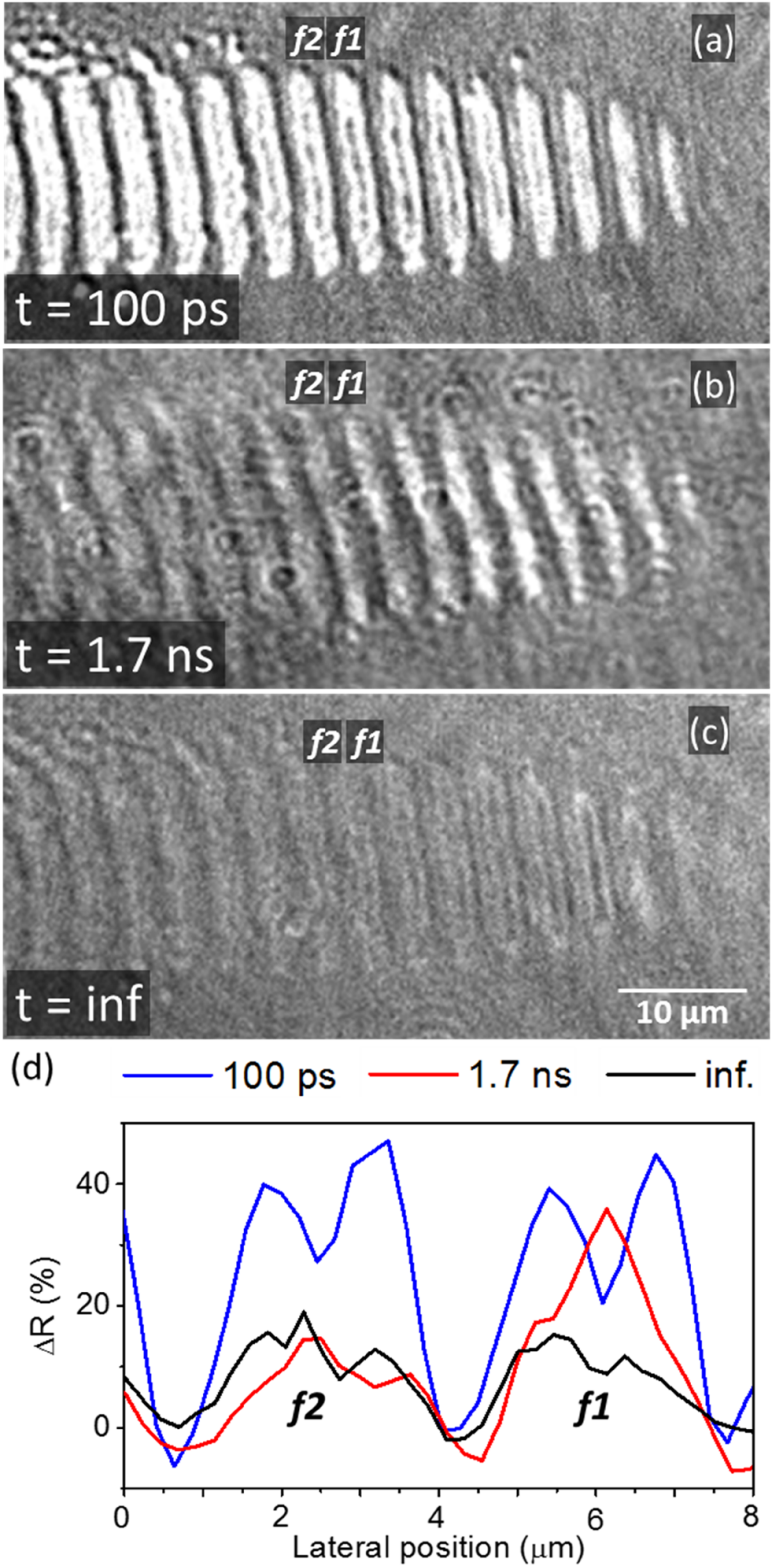
(**a**–**c**) Time-resolved optical micrographs of *Λ* ^*s*+^ fringe structures being written upon irradiation of a moving Si sample (towards the left) with fs laser pulses at oblique incidence (*θ* = *52°*). The delay times are indicated in the figures and *t* = *inf*. refers to a delay of several seconds. (**d**) Horizontal profiles through the fringes *f1* and *f2* indicated in (**a**–**c**), quantifying the relative reflectivity change ΔR.

A detailed study about the complete formation dynamics of fringe structures can be found in our recent work^15^. In the present work, we want to focus on a different aspect and illustrate it with new experimental data. We have used the moving-spot modality of fs microscopy^15^, based on recording time-resolved images while the sample is translated using a step size equal to the fringe period. Laser irradiation was performed at *θ* = *52°* and illumination with a fs probe pulse at *θ* = *0°* (c.f. Section “Methods”). Figure 10(a) shows fringes near the writing front at a delay *t* = *100* *ps* after the arrival of a pump pulse. It can be seen that the fringes show the characteristic high reflectivity of liquid Si, well above that of the fringes in (c) several seconds after, which corresponds to amorphous Si. At longer delays (Fig. 10(b), *t* = *1.7* *ns*) the reflectivity decreases in the left part of the image, indicating solidification into the amorphous phase. In the right part, exposed to the peak fluence of the laser pulse, the reflectivity remains rather high. The corresponding profiles shown in Fig. 10(d) reveal the transition region in which fringe 1 (*f1*) is still in the liquid phase at *t* = *1.7* *ns* whereas fringe 2 (*f2*) has already solidified, featuring the same reflectivity level as the fringe at *t* = *inf*.

An important conclusion that can be drawn from this data is that the formation of amorphous-crystalline LIPSS upon irradiation of a moving sample involves *melting only certain regions*, specifically those which eventually end up amorphous. Melting is not induced in the regions between amorphous fringes.

### High quality large-area gratings

**Figure 11:**
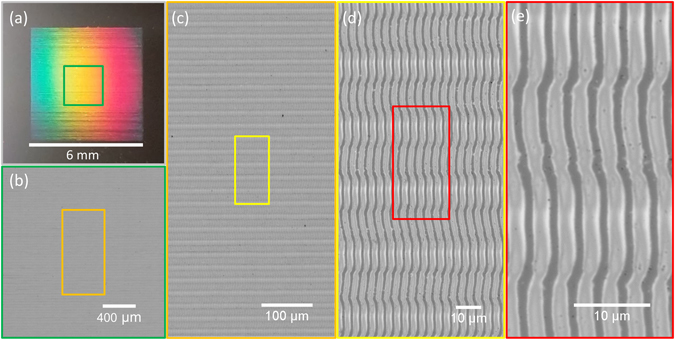
Large area grating (6 × 6 mm) written by scanning the sample in two dimensions. (**a**) Image recorded with a smartphone camera upon angled illumination with white light, illustrating the efficient and homogeneous diffraction. (**b**–**e**) Optical micrographs recorded with different magnifications M upon illumination with a blue LED at normal incidence. (**b**) M = 2.5× (**c**) M = 10× (**d**) M = 50× (**e**) M = 150×. The regions over which was zoomed in for each magnification are marked by rectangles. The writing process was performed at a laser repetition rate *f* = *250 Hz*, *F* = *160 mJ/cm* ^*2*^ and the sample was scanned continuously at *v* = *2300 µm/s* along the horizontal direction, with a discrete vertical line separation *d* = *13 µm*.

From an application point of view, it is vital to develop efficient strategies for fabricating large-area gratings in Si. Several works report on the fabrication of extended areas of ablation-based LIPSS in silicon, both for low-spatial-frequency^26^ and high-spatial-frequency^27^ LIPSS. A recent attempt to fabricate extended gratings of amorphous fringes using a Galvo-scanner system at normal incidence only provided just moderate results^16^. In that case, the connection between fringes of adjacent line scans was compromised by the resolution of the scan head and notably affected the quality of the grating structures produced, featuring signs of ablation. Here, we have performed sample scanning to overcome this limitation. Moreover, in order to demonstrate that this approach is compatible also with the tuneability of the grating period via angled incidence, we have written a large area grating of *Λ* ^*s*+^ fringes at *θ* = *52°*. Figure 11 shows the resulting 6 × 6 mm^2^ grating. An indication of the high quality is its performance in diffraction upon illumination with white light, featuring homogeneously distributed rainbow colors without distortion. Upon inspection under an optical microscope with illumination at 460 nm, the spatial homogeneity over large areas is best appreciated at low magnifications (2.5×), whereas individual fringes and their connections are best appreciated at higher magnifications. Astonishingly, the continuous extension of the fringes of adjacent horizontal line scans occurs over the entire scan field, leading to single fringes which are 6 mm long, without interruption. The alternating slight shift and bending of fringes at the scan line transition is caused by the scan strategy employed, in which odd scan line numbers were written first, followed by the writing of all even line numbers.

## Conclusions

In this paper, we have fabricated amorphous-crystalline LIPSS in crystalline Si using irradiation with multiple femtosecond laser pulses. Upon static irradiation, the interference of incident laser light with a scattered or propagated surface wave leads to the formation of LIPSS with only moderate regularity. Upon moving the beam over the sample surface, these structures become highly periodic. Micro-Raman spectroscopy and fs microscopy studies demonstrate that these fringe structures are formed upon local melting and rapid solidification into the amorphous phase. Topography studies show that the formation process does not involve surface ablation but minor evaporation, which increases as a function of pulse number. The local thickness of the amorphous layer of a single fringe can be determined by means of an analysis of the reflectivity, which allows the determination of the fringe cross section. We demonstrate that the optical properties, thickness, surface depression and lateral dimensions of the fringes can be controlled by adjusting experimental parameters. Moreover, the period can be controlled by changing the angle of incidence and inverting the scan direction, covering a range of *Λ* = *410* *nm* − *13* *µm* by angle tuning up to *θ* = *70°* for a single laser wavelength. The experimentally obtained period matches well a surface scattering model, without the need to involve surface plasmon polaritons. We show that extremely homogeneous, large-area gratings can be fabricated, featuring continuously extended fringes with a width of micrometers and a length of several millimeters. The simplicity and high flexibility of this structuring strategy should find applications in numerous fields, including optics, nanoelectronics, and mechatronics. Moreover, it is expected to work with materials other than silicon, in which a phase transition can be trigged by laser pulses.

## Methods

**Figure 12:**
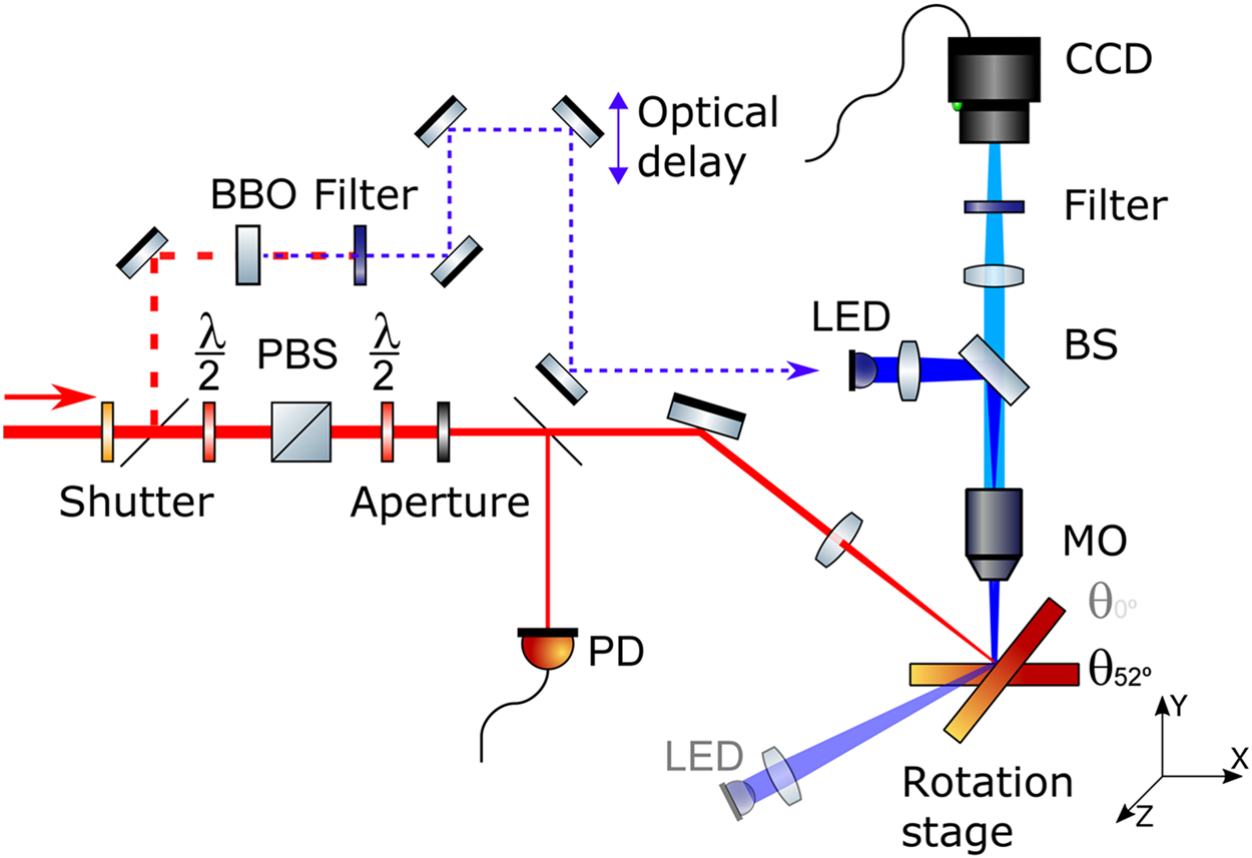
Experimental setup for laser fabrication and *in-situ* characterization of amorphous-crystalline structures in Si including *in-situ* monitoring. BS beamsplitter, MO microscope objective, PD photodiode, PBS polarizing beamsplitter, BBO frequency doubling crystal. The dashed-line optical path corresponds to fs-pulsed probe illumination, alternative to the CW LED illumination for visualization.

The experimental configuration is schematically shown in Fig. 12. The laser system used for irradiation is a Ti:sapphire femtosecond amplifier providing pulses of 120 fs FWHM (Full Width at Half Maximum) at 800 nm central wavelength with a repetition rate of 100 Hz. A mechanical shutter enables the selection of single pulses or pulse bursts. The pulse energy is adjusted by a combination of a half-waveplate and a polarizing beam splitter cube. A second half-waveplate is used to set the polarization of the pulse to p-polarized at the sample plane. The beam passes through a beam shaping circular aperture with a diameter *Ø* before being focused by a lens with focal length *f* at the sample surface, at an angle of incidence *θ*. The angle of laser incidence is defined with respect to the optical axis of the microscope. Irradiation experiments were performed at *θ* = *52°* (sample surface normal to the microscope axis) and *θ* = *0°* (sample surface normal to the laser beam axis). The spot size of the laser at the sample surface could be changed by using lenses with different focal lengths (*f* = *80* *mm* or *f* = *150* *mm*) and/or using different diameters of the beam shaping circular aperture (*Ø* = *3.5* *mm* or *Ø* = *5* *mm*). For all cases the intensity distribution is Gaussian (*1*/*e* ^*2*^ − diameter d measured experimentally)^28^. For oblique incidence, the spot size was elliptic with the long axis *dx* lying in the plane of incidence. The standard irradiation conditions employed in most cases were *f* = *150* *mm* and *Ø* = *3.5* *mm*, leading to a spot diameter of *d*_*y*_ = *59* *µm* and *d*_*x*_ = *103* *µm*.

The exposed region of the sample is monitored *in-situ* with an optical microscope, consisting of a microscope objective (MO, 20×, *N.A*. = *0.42*), a tube lens, and a CCD camera, protected by a filter to block the pump light. Illumination was performed in reflection using LEDs (400 nm wavelength) at different positions according to the used angle of incidence, as illustrated in Fig. 12. For specific experiments at *θ* = *52°*, fs-pulsed laser illumination was employed upon irradiation in order to study the dynamics of laser-induced melting and solidification. To this end, a fraction of the pump laser beam was split off, frequency-doubled in a BBO crystal to 400 nm and used to illuminate the sample at certain delay times after arrival of the pump pulse. The delay between pump and probe was controlled by means of a motorized optical delay line.

The sample was a comercial 〈100〉 oriented crystalline silicon wafer with p-doping (boron, resistivity 1–5 Ω cm). It was mounted on a rotation stage in order to select the irradiation angle *θ* and on a motorized three-axis translation stage to position the sample or move it at a user-defined constant speed. After irradiation, the laser-exposed regions were characterized using a variety of techniques. Optical microscopy was performed employing a Nikon Eclipse Ti inverted microscope with a 100× dry objective lens (*N.A*. = *0.9*) and LED illumination at 460 nm (except for Fig. 5, where 400 nm and 760 nm were used as additional wavelengths), yielding a maximum lateral resolution *R*_*xy*_ < *300* *nm*. The surface topography of the structures was measured with an optical profiler (Sensofar Plμ 2300) based on white light interference, using a 50× objective lens (*N.A*. = *0.55*). While this technique provides only a moderate lateral resolution (*R*_*xy*_ = *600* *nm*) the vertical resolution of the system is *Rz* $\ll$ *1* *nm*. The surface morphology of the structures was characterized using scanning electron microscopy (SEM, Hitachi S-4800). Micro-Raman spectroscopy was employed to investigate the structure and Raman mapping was applied to check the spatial distribution of both the amorphous and crystalline areas. To this end, a Renishaw InVia instrument was employed, using an excitation laser at 532 nm in order to obtain intense bands from both the amorphous and crystalline phase of Si. Single point spectra were obtained in backscattering configuration by using a laser power at the sample of 0.1 mW focused to a spot diameter of *d* = *1* *µm* and using an objective 100×, *N.A*. = *0.85*. Raman maps of 25 × 25 µm^2^ were recorded by using the Stream-LineTM mode, providing 600 spectra in 10 min. for an overall laser power of 25 µW extended over a line focus of 1 × 12 µm^2^.
