# Author Correction: Coherent scatter-controlled phase-change grating structures in silicon using femtosecond laser pulses

**DOI:** 10.1038/s41598-018-24091-x

**Published:** 2018-04-12

**Authors:** Yasser Fuentes-Edfuf, Mario Garcia-Lechuga, Daniel Puerto, Camilo Florian, Adianez Garcia-Leis, Santiago Sanchez-Cortes, Javier Solis, Jan Siegel

**Affiliations:** 1 Laser Processing Group, Instituto de Óptica, IO-CSIC, Serrano 121, 28006 Madrid, Spain; 2 Instituto de Estructura de la Materia, CSIC, Serrano 121, 28006 Madrid, Spain

Correction to: *Scientific Reports* 10.1038/s41598-017-04891-3, published online 04 July 2017

The Acknowledgements section in this Article is incomplete.

“M.G.-L. thanks the Spanish Ministry of Education for a FPU fellowship. This work was supported by the LiNaBioFluid project (H2020-FETOPEN-2014-2015RIA, grant 665337) of the European Commission; and the research grant (TEC2014-52642-C2-1-R) from the Spanish Ministry of Economy and Competitiveness.”

should read:

“M.G.-L. thanks the Spanish Ministry of Education for a FPU fellowship. This work was supported by the LiNaBioFluid project (H2020-FETOPEN-2014-2015RIA, grant 665337) of the European Commission; and the research grants (TEC2014-52642-C2-1-R and FIS2014-52212-R) from the Spanish Ministry of Economy and Competitiveness.”

